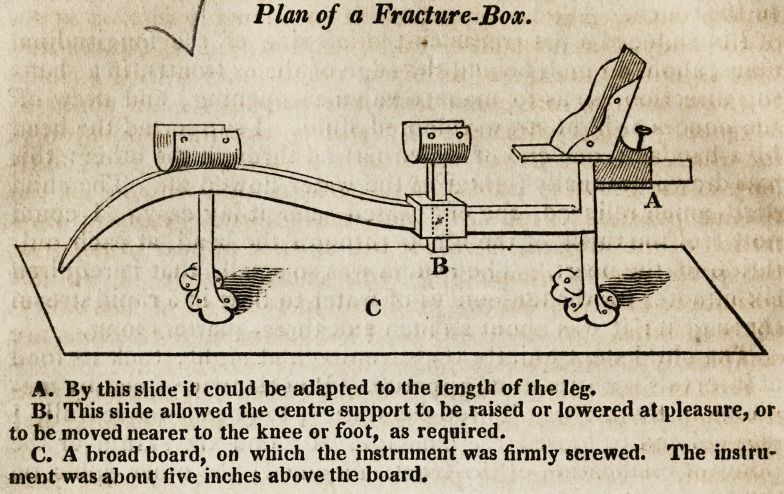# Surgical Cases

**Published:** 1825-09

**Authors:** Robert R. Gray

**Affiliations:** Licentiate of the Royal College of Surgeons in Ireland, &c. &c.


ART. V.—*Surgical Cases.* By ROBERT R. GRAY, Licentiate of the Royal College of Surgeons in Ireland, &c. &c.

1. *A Case of Hydrocephalus in a Child of nine Months old, in which the Head was tapped three times.*

I WAS called to see a child, the son of a poor man, in the month of November last, in which I recognised a former patient. I had seen it eight months before, under the following circumstances, and with the following diseases and appearances: viz.—spina bifida; head somewhat larger than natural; the legs, instead of bending back on the thighs, bent forward; it had no patellæ; feet turned in, and were what is commonly termed web feet. The child otherwise was the picture of health.

I ordered moderate pressure to the tumor on the spine, which in some time had the effect of consolidating it, without appearing to affect the child's health. I did not see it then for some time. When I was called to see it again, the child was nine months old,—that is, eight months since I saw it first. The child was now much altered; the head much increased in size, the forehead projecting; the face small and triangular; the eyes sunk, and encircled with a yellowish matter, partly dried; appetite voracious; bowels confined; makes water naturally; has a constant whining cry; sometimes screams out, as if in violent pain. The scalp interspersed with spots, from which flowed a quantity of water, which I wished to increase, and ordered a soft poultice for the purpose; which had not the effect intended, and was discontinued after a few days. Ordered some purgative medicine every second day, which did no other good than to free the bowels.

I now saw the child had no chance of living, and that it was a fair subject for experiment, which could not be called cruel, the child being always relieved by the operation. I determined to tap the head, which at this time measured as follows:—Largest circumference, twenty-seven inches; from the tip of one ear to the other, across the top of the head, eighteen inches; from the same points, round the occiput, seventeen inches; from the base of the nose to as near as I could go to the atlas, nineteen inches; separation in the coronal suture of the bones, two and a half inches; in the sagittal suture, three inches: os frontis ossified in the centre. Scalp thinly covered with hair, and scabby. Pulse intermitting, so weak and quick I could not count it; breathing regular while lying quiet; head feels warm to the touch.

I introduced a flat trocar on the left side of the longitudinal sinus, about an inch behind the edge of the os frontis, in a slanting direction, so as to make a valvular opening, and drew off ten ounces of light straw-coloured fluid. I supported the head by a bandage, one end of which passed through the other: this was drawn gradually tighter as the water flowed off. The child very much relieved, the cry ceased, and it lay easy. I could now feel the thrill of the blood through the head, at each pulsation of the heart. The canula was so small, that it required six minutes for the ten ounces of water to flow in a rapid stream through it: it was about an inch and three-quarters long.

The child slept quietly that evening and night; took its food at intervals with its usual appetite. Bowels twice freed by medicine in the night. Three days after, the head was so full, I was obliged to loosen the bandage. I found the skin over the point of ossification of the frontis inflamed; the other spots on the head irritated by the pressure of the bandage.

On the tenth day after the first operation, I tapped it again, and took off sixteen ounces of rather a darker fluid. The child experienced the same relief as before: treated as before.

Eleven days after this, tapped it again, and drew off fourteen ounces of fluid, such as the first; and the child appeared equally relieved.

I could not now see it for about fourteen days that I was otherwise employed, and on my return I found the child had been dead about five days. I, of course, had no opportunity of examining the head.

2. *A Case of very short Funis Umbilicus, whereby the Child was nearly lost.*

On the child's being born, it was removed to about six inches from the mother: it made a strong inspiration, but appeared unable to expel the air from the lungs. I thought there might be mucus in the mouth, which I attempted to remove with a corner of the towel, but the child got no relief; it appeared greatly oppressed. I called for some spirits to rub the chest with, conceiving that it might be want of irritability in the muscles of the chest and abdomen which prevented their action; and in the mean time I endeavoured to ascertain if any other cause existed, when I found the funis very much on the stretch, and the parietes of the abdomen drawn into a funnel-shape. I immediately placed the child nearer to the mother, to allow the abdominal muscles and diaphragm to act, and the child cried loudly. I separated the child from the mother, and it recovered.

---

*Plan of a Fracture-Box.*

A. By this slide it could be adapted to the length of the leg.

B. This slide allowed the centre support to be raised or lowered at pleasure, or to be moved nearer to the knee or foot, as required.

C. A broad board, on which the instrument was firmly screwed. The instrument was about five inches above the board.

The above is a rough sketch of an instrument I found extremely useful in compound fracture of the leg, when the man would not submit to amputation. The weather was warm, and I found it very difficult to dress the wound at the under part, without disturbing the broken ends; and to leave it one day without being dressed, I should have had it full of maggots. By using this instrument, I could look under the sore at pleasure, without disturbing the bones so much as I otherwise must have done. I padded the rest for the knee and foot well, then laid in the leg, and raised the centre support so as to let both broken ends lie on it. At the foot part may be seen sides, which, when closed as they appear, and fastened with tape, prevented the foot from turning. The foot was firmly bandaged into this, as was the knee into its rest; the leg was then stretched as much as it would bear, by the foot part being removed along the bar, and there fastened with the screw over A. When I dressed the leg, I let down the centre support, and slided it up a little toward the knee; then raised it so as to support the heaviest end of the fractured part; by which means the sore was entirely exposed. When dressed, I replaced the centre support under the sore, with a dry cushion on it. While moving the centre with one hand, I supported the leg with the other.

*Galway; April* 14, 1825.

## Figures and Tables

**Figure f1:**